# Structural complexity governs seagrass acclimatization to depth with relevant consequences for meadow production, macrophyte diversity and habitat carbon storage capacity

**DOI:** 10.1038/s41598-019-51248-z

**Published:** 2019-10-10

**Authors:** Susana Enríquez, Irene Olivé, Napo Cayabyab, John D. Hedley

**Affiliations:** 1 Laboratorio de Fotobiología, Unidad Académica de Sistemas Arrecifales Puerto Morelos, Universidad Nacional Autónoma de México, Apto. Postal 13, 77500-QR Cancún, Mexico; 2 Present Address: School of Geographical and Earth Sciences, Gregory Building Rm419, University of Glasgow, Glasgow, G12 8QQ UK; 3 Numerical Optics Ltd., Belmont House, Witheridge, Tiverton, Devon EX16 8AA UK

**Keywords:** Ecophysiology, Light responses, Plant ecology

## Abstract

Analyses of the integrated seagrass response to depth support the previously documented low plasticity and consistent shade-adapted leaf physiology of a habitat-builder that dominates well-illuminated reef environments. Two structural responses, “canopy-opening” and “below-ground-mass-depletion”, govern the photoacclimatory response and facilitate, respectively, light penetration within the canopy and functional adjustments in whole-plant carbon balances. Conversely, “canopy-closing” may also explain dense canopies formed close to the waterline, as they provide shade and photoprotection to a susceptible leaf physiology under high-light. Canopy light attenuation is primarily regulated by the leaf area index (LAI), which is governed by changes in shoot size and density. Shoot density diminishes non-linearly with depth, while shoot size increases to a maximum followed by a decline. The initial increase in shoot size, which resembles a self-thinning response, increases LAI and meadow production in shallow depths. These seagrass structural adjustments have relevant ecological implications. Canopy-thinning allows macrophyte diversity to increase with depth, while seagrass production and carbon storage diminish exponentially, and are maximal only in a shallow coastal fringe. The results support the universality of plant self-thinning, from phytoplankton to complex canopies, likely the consequence of simple physical laws related to light limitation and pigment self-shading within photosynthetic structures and communities.

## Introduction

Seagrasses build highly productive coastal habitats across all continents except Antarctica^1,2^. Despite only occupying 0.1% of the global ocean surface, seagrass meadows are considered important oceanic sinks for atmospheric CO_2_^3^, being responsible for the sequestration of 20% of the global organic carbon present in marine sediments^4^. High burial rates and slow organic carbon decomposition in the sediment^5^ are consistent with this interpretation. However, a large variability in carbon stock and accumulation rates among species and habitats has been documented^6^, as well as a wide spatial and temporal variation in seagrass standing biomass and daily leaf production^2,7^. For example, in oligotrophic reef environments seagrasses often colonize coarse carbonate sediments where their capacity to retain the organic carbon produced is small^8,9^. For the climax species in the Caribbean and Gulf of Mexico, *Thalassia testudinum*, a variation of up to 100-fold in standing biomass has been documented^10^. Nutrient limitations such as nitrogen^11^, phosphorus^12^, or iron^13^, have been proposed as the main limiting factors for seagrass productivity in reef environments; while high-light stress^14,15^, and fresh-water inputs^16^, have been also suggested to affect seagrass growth and production.

Light is a central parameter in the regulation of plant growth and production, and consequentially affects the capacity of seagrass meadows to store organic carbon^17^. The direct effect of light on seagrass production and habitat structure, however, has not yet been addressed in the context of carbon storage, despite its critical role in seagrass capacity to sequester organic carbon at global scales. Light attenuation with depth is a major environmental gradient for benthic macrophyte communities, and is responsible for physiological and structural changes leading to reduction in seagrass growth and production^18–21^. The magnitude of the decline in production with depth depends on both light attenuation within the water column and also on the species ability to photoacclimatize to the reduction in light. In general, large habitat-building seagrasses have shown greater plant and canopy structural changes with depth, but only limited physiological variability. This has been documented for the leaves of *Thalassia testudinum*^22^, *Posidonia oceanica*^19^, and *Posidonia sinuosa*^21^. Olesen *et al*^19^. interpreted this finding in terms of the capacity of these large species to modify the internal canopy light field, concluding that this structural regulation of the internal canopy illumination could explain the small leaf-level physiological response to depth. However, Dennison & Alberte^23^ and Cayabyab & Enríquez^15^ have also provided experimental evidence of physiological limitation in the photoacclimatory leaf response of two large seagrasses. A possible explanation of the low physiological plasticity of the leaves of *T. testudinum*^15^ is that this is the result of an evolutionary shade-adapted constraint of the group as a whole^24^. Later evidence derived from the characterization of the high-light response of the leaves of *T. testudinum* supports this interpretation as some characteristics of a shade-adapted physiology still persist (i.e., small xanthophyll-pool size, slow non-photochemical-quenching (NPQ) induction rates and extremely low de-epoxidation-state-DPS values^25^) despite the fact that maximum values for NPQ are similar to those achieved by terrestrial sun leaves^26,27^.

Globally, large differences among seagrass species in their photoacclimatory response have been documented^15,16,18–23,28–30^, as well as in leaf photosynthetic performance^14,31,32^, but few studies have investigated the effect of the canopy light field on this variation. Empirical measurements^14,33^ and bio-optical models^34,35^ have described a large variation in irradiance within seagrass canopies, and its strong dependence on the Leaf Area Index (LAI) and shoot density^33^. Downwelling light attenuation coefficients (*K*d, m^−1^) within seagrass canopies are estimated to be one order of magnitude greater than water-column coefficients^14,33,35^. Therefore, as seagrass leaves elongate from their basal meristem and occupy successively higher positions within the canopy, they have to respond to an increasing and fluctuating light environment. The relevance of within-canopy variation in irradiance on leaf photoacclimatization is supported by the documented differences along the leaf in pigment content, leaf absorptance, maximum photochemical efficiency (*Fv/Fm*), photoprotection capacity (NPQ), and in the pattern of PSII recovery through D1 protein synthesis^14,25^. Therefore, the potential combined effect of the changes in light levels due to the water column with the structural variation of the canopy, suggests that canopy structure must be a fundamental component of the photoacclimatory response of seagrasses to depth.

In this study, we aimed to characterize the photoacclimatory response to depth of *Thalassia testudinum*, Banks ex König, within the context discussed above, and thus, focussing the attention on both leaf level physiology and canopy structure. The characterization was however performed at multiple levels of plant and community organization: from changes in leaf physiology and pigmentation, to the analysis of the variation in the carbon allocation patterns of the seagrass, and changes in habitat structure and macrophyte diversity. *T. testudinum* is the main habitat-builder in the Caribbean and Gulf of Mexico, hence, this characterization has implications relevant for production and carbon storage at global scale.

## Results

**Figure 1:**
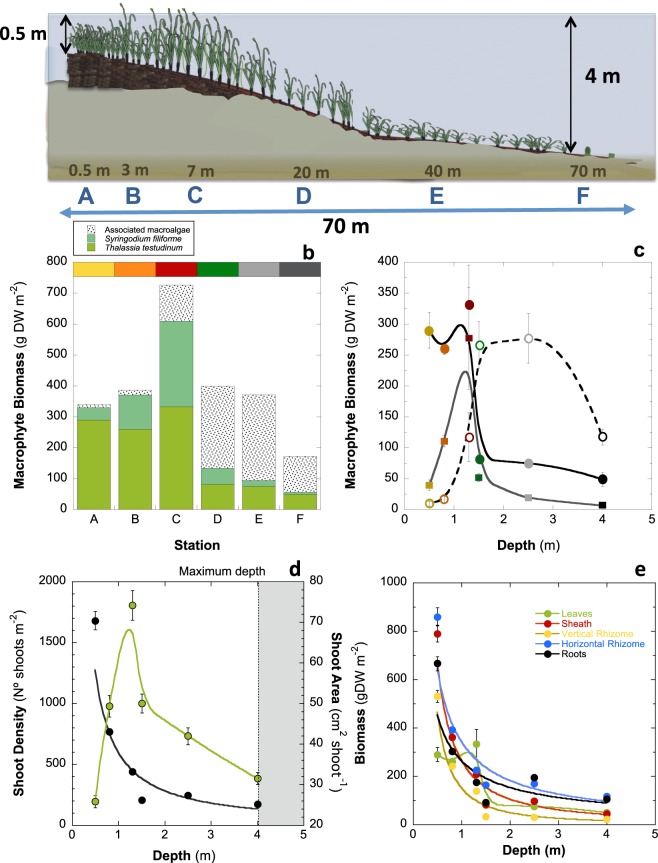
Structural variation of the seagrass meadow with depth. (**a**) Diagram describing the structural changes and the location of each sampling site; (**b**) cumulative variation in macrophyte abundance (g dw m^−2^); (**c**) variation (avg ± SE) of *Thalassia testudinum* biomass (coloured solid circles) with depth, *Syringodium filiforme* (squares) and macroalgae (white circles); (**d**) variation in shoot density (#shoots m^−2^) dark circles) and shoot size (cm^2^ shoot^−1^) green circles) of *T. testudinum* with depth; and (**e**) biomass variation of each plant component of *T. testudinum*: leaves (green), sheaths (red), vertical rhizome (yellow), horizontal rhizome (blue) and roots (black) with depth. The grey area in plot 1d indicates the maximum depth of the reef lagoon, and thus, the prediction limit for the quantitative models.

*Thalassia testudinum* was the dominant species in all sites and especially at the shallowest stations, which showed the highest abundance (Fig. 1). Along the transect an up to 5-fold seagrass decline was observed after station C, although the narrow-leaved seagrass *Syringodium filiforme* showed a peak in abundance at that site, after which the diversity of macroalgae in the community was significantly enhanced with depth (Fig. 1b,c; SI-Table-S1). Macroalgal presence was highest at sites D and E, and very low at sites A and B.

The decline in the *T. testudinum* above-ground mass was mainly due to a non-linear reduction in shoot density:

1 $$\begin{array}{l}{\rm{Shoot}}\,{\rm{density}}=[159\pm 29]+[415.6\pm 41.5]\cdot {{\rm{Depth}}}^{(-1.87\pm 0.13)}\\ ({{\rm{R}}}^{2}=0.99;\,{\rm{P}} < 0.001)\end{array}$$

Shoot size showed a more complex pattern of variation with depth manifested as an initial linear increase until a maximum value was reached, followed by a non-linear decline from that maximum (Fig. 1d). The variability in shoot density was almost twice as large as the variability in shoot size (respective coefficients of variation, C.V., of 109% and 57.5%; Table 1). The other structural descriptors presented C.V. between 24.6 and 134.4% (Table 1).

**Table 1:** Mean ± SE values of the physiological and structural traits of *Thalassia testudinum* characterized for six sites located along a continuous meadow of the reef lagoon, from the shallowest (A) to the deepest site (F). Maximum photosynthesis, P_max_, and light-enhanced respiration, R_L_ (µmol O_2_ cm^−2^ h^−1^); photosynthetic efficiency, α, (mol O_2_ evolved per mol incident quanta); saturation, Ek, and compensation, Ec, irradiances (µmol quanta m^−2^ s^−1^); quantum efficiency, Φ_max_, (mol O_2_ evolved per mol quanta absorbed) and minimum quantum requirements, 1/Φ_max_, of leaf photosynthesis (mol quanta absorbed per mol O_2_ molecules evolved); Absorptance (% of incident PAR absorbed); maximum photochemical efficiency of photosystem II, *Fv/Fm*; and chlorophyll content, Chl[a + b] (mg m^−2^). The coefficient of variation (C.V.) and the number of samples determined for each parameter (n) are also shown. Structural traits: shoot density (# m^−2^); Shoot size (cm^2^ shoot^−1^); Leaf Area Index-LAI; below- and above-ground biomass of *T. testudinum* (g dw m^−2^) and leaf mass ratio, LMR (% leaf biomass).

Parameter	Site A	Site B	Site C	Site D	Site E	Site F	C.V.	n
P_max_	0.83 ± 0.07	1.10 ± 0.09	0.84 ± 0.08	1.25 ± 0.10	1.12 ± 0.11	1.10 ± 0.11	38.3%	93
R_L_	0.032 ± 0.015	0.242 ± 0.041	0.383 ± 0.072	0.375 ± 0.069	0.259 ± 0.033	0.283 ± 0.043	76.5%	93
α	0.029 ± 0.003	0.040 ± 0.003	0.041 ± 0.003	0.048 ± 0.004	0.042 ± 0.004	0.043 ± 0.004	33.3%	80
Ek	88.0 ± 8.6	84.3 ± 11.4	59.9 ± 7.2	82.0 ± 12.8	78.3 ± 9.9	79.1 ± 11.6	51.8%	80
Ec	2.8 ± 0.4	18.4 ± 3.0	19.2 ± 3.0	19.3 ± 3.4	18.2 ± 1.9	18.4 ± 2.5	63.2%	93
Absorptance	48.9 ± 1.27	55.4 ± 2.26	51.9 ± 1.10	57.1 ± 0.78	50.2 ± 0.89	52.5 ± 0.96	10.8%	50
Φ_max_	0.065 ± 0.005	0.068 ± 0.004	0.080 ± 0.008	0.076 ± 0.008	0.081 ± 0.006	0.071 ± 0.009	33.6%	93
1/Φ_max_	15.5 ± 1.1	14.7 ± 0.9	12.6 ± 1.2	13.1 ± 1.4	12.4 ± 0.9	14.1 ± 1.7	35.3%	93
*Fv/Fm*	0.788 ± 0.008	0.833 ± 0.002	0.812 ± 0.003	0.813 ± 0.002	0.809 ± 0.002	0.813 ± 0.002	2.3%	88
Chl [a + b] content	53.6 ± 3.8	102.3 ± 10.3	87.8 ± 4.8	103.8 ± 4.98	87.2 ± 3.9	94.1 ± 3.96	41.4%	269
Shoot Density	1678.9 ± 75.6	767.3 ± 25.2	440.6 ± 22.5	204.2 ± 17.1	244.6 ± 17.2	170.3 ± 13.6	109%	87
Shoot size Area (cm^2^)	25.84 ± 1.46	49.33 ± 2.71	74.17 ± 3.59	50.05 ± 2.31	41.99 ± 2.08	31.49 ± 1.56	57.6%	463
LAI	4.3 ± 0.2	3.8 ± 0.1	3.3 ± 0.2	1.02 ± 0.08	1.03 ± 0.07	0.53 ± 0.04	83.5%	87
Belowground Biomass	2846.4 ± 128.2	1300.8 ± 42.9	747.4 ± 38.2	373.4 ± 31.3	494.2 ± 34.7	288.7 ± 23.1	106.2%	87
Aboveground Biomass	290.1 ± 28.6	259.8 ± 9.3	332.2 ± 63.3	81.7 ± 7.1	74.9 ± 4.5	48.9 ± 11.4	78.9%	35
LMR	9.25%	16.7%	30.8%	17.96%	13.2%	14.5%	21%	87

**Figure 2:**
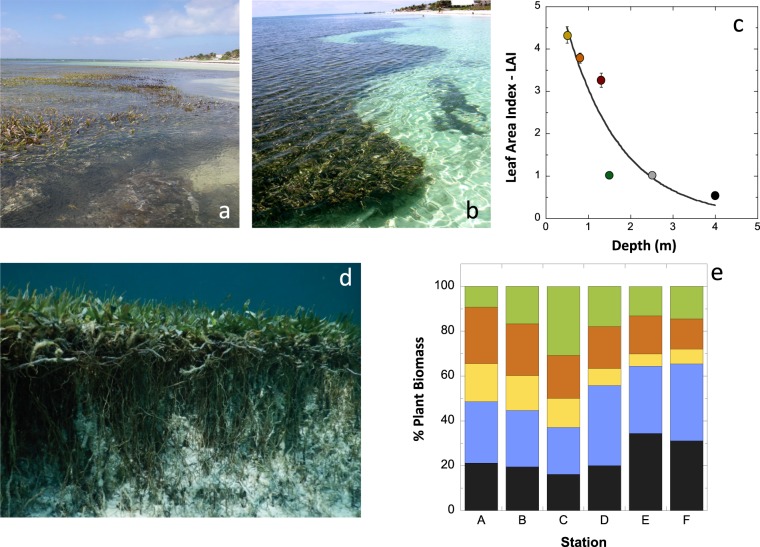
Images of the edge of the seagrass meadow at the shore and of the variation of canopy structure (LAI) and seagrass biomass partitioning with depth. Plots (**a**, **b** and **d**) are different images of the edge of the seagrass meadow at the shore; (**c**) describes the variation of the leaf area index (LAI) with depth; and (**e**) describes the cumulative variation of biomass partitioning with depth for *Thalassia testudinum*. Colours represent: the different sites (A to F) according to figure 1 for plots (**b** and **c**); and leaves (green), sheaths (orange), vertical rhizomes (yellow), horizontal rhizomes (blue) and roots (black) for plot (**e**).

LAI reached maximum values on a shallow coastal platform formed by the accumulation of dense stands of belowground biomass and sediment (sites A-C; Figs 1 and 2), and showed an abrupt decline at the end of this platform, between stations C and D (Fig. 2c). Towards the shore, the platform ended in a 0.3–0.5 m scarp, which exposed seagrass rhizomes to the water, while towards the lagoon the platform showed a less abrupt profile (Figs 1 and 2). The reduction in aboveground-biomass was only visible for stations located beyond the platform, whereas the non-linear reduction of the belowground biomass was observed from the shore (Fig. 1c,e). Significant variation in seagrass mass allocation to different components of the plant was observed with depth, due to differences in the pattern of change of each plant module (Fig. 2e). The biomass of sheaths and vertical rhizomes experienced a progressive 1.5-fold reduction over depths from 0.5 to 4 m, while horizontal rhizomes and, especially, root biomass significantly increased at the deepest stations. Due to this heterogeneous variation, site C showed the highest proportion of seagrass biomass allocated to photosynthetic tissues (LMR = 30%), while the other five sites presented LMR values below 20% (Table 1). The rapid LAI decline observed between station C and D coincided with changes in mass allocation from vertical to horizontal rhizome growth (Fig. 2).

### Canopy light field

**Figure 3:**
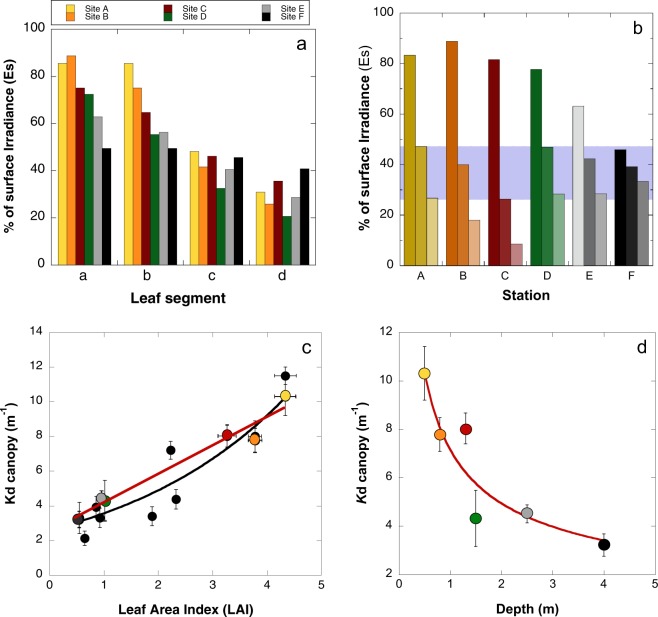
Canopy light attenuation (Kd_canopy_) with depth. Colours of the histograms and circles are the same used in Fig. 1 for describing each site. (**a**) Histogram describing the variation of the percentage of surface irradiance (%Es) at four leaf levels (“a”-“b”-“c”-“d”) of each canopy (from A shallowest, to F deepest); (**b**) changes in %Es at the top of the canopy, middle and sediment level, blue highlights the values of %Es achieved at the middle level of each canopy; (**c**) variation in meadow Kd_canopy_ (m^−1^) as a function of changes in LAI, red line describes a least-square regression fit (R^2^ = 0.96; p < 0.001) for the data generated in this study (coloured circles) and the black line is an exponential fit (R^2^ = 0.89; p < 0.01) comparing pooled data (n = 15) from values reported by Enríquez and Pantoja-Reyes^33^ (black circles); and (**d**) variation of the Kd_canopy_ (m^−1^) of each site with depth, the continuous red line describes the exponential fit (R^2^ = 0.85; p < 0.05).

Estimated *K*d_canopy_ values were one order of magnitude greater than those of the water column, indicating substantial leaf self-shading even within a relatively low canopy (<20 cm high; Table 2). The largest *K*d_canopy_ was found at the shallowest station, and the smallest at the deepest site. Using the estimated *K*d_canopy_ and data on canopy height and maximum leaf length, we estimated the percentage of surface irradiance (%*E*s) reaching each leaf segment (Fig. 3a). Estimated %*E*s at the top of canopies differed 2-fold between the shallowest and deepest stations, while vertically within the individual canopies, light levels varied up to 6-fold (Table 2, Fig. 3a). When comparing leaf segments, the apical portions (“a” and “b”) differed among stations by ~40% *E*s, on average, while only a ~15% variation was observed for the middle and middle-basal portions (“c” and “d”). Notably, light exposure was very similar across all canopies for the leaf segments located in the middle of the canopy (Fig. 3b; Table 2).

**Table 2:** Canopy height and downwelling attenuation coefficients (Kd, m^−1^) determined for the water column and for each seagrass canopy throughout a depth gradient in the reef lagoon of Puerto Morelos (A-F). The variation in the percentage of surface irradiance (%Es) at the top, middle and bottom of each canopy is also shown.

Station	Depth (m)	Canopy height (cm)	*K*dwater (m^−1^)	*K*dcanopy (m^−1^)	% Es Top Canopy	% Es Middle Canopy	% Es Bottom Canopy
A	0.5	11.0 ± 0.00	0.47 ± 0.03	10.31 ± 1.11	83.25	47.22	26.78
B	0.8	20.6 ± 1.17	0.20 ± 0.01	7.78 ± 0.70	88.80	39.85	17.88
C	1.3	28.2 ± 0.88	0.20 ± 0.01	8.03 ± 0.64	81.58	26.30	8.48
D	1.5	23.4 ± 0.84	0.20 ± 0.01	4.31 ± 1.16	77.64	46.86	28.28
E	2.5	17.7 ± 0.61	0.20 ± 0.01	4.50 ± 0.37	62.83	42.22	28.37
F	4	9.9 ± 0.49	0.20 ± 0.01	3.22 ± 0.46	45.83	39.07	33.30

Differences in shoot density explained 78% of the *K*d_canopy_ variation, while differences in LAI explained 96% (Fig. 3c). When comparing *K*d_canopy_ estimations with the values documented by Enríquez & Pantoja-Reyes^33^, LAI was the best descriptor of seagrass *K*d_canopy_ variation (Fig. 3c), while a positive effect of shoot size was also found (covariance-test, t-student; p < 0.01) on the slope of the linear association between shoot density and *K*d_canopy_ (R^2^ = 0.78; P < 0.05).

### Leaf photoacclimation

**Figure 4:**
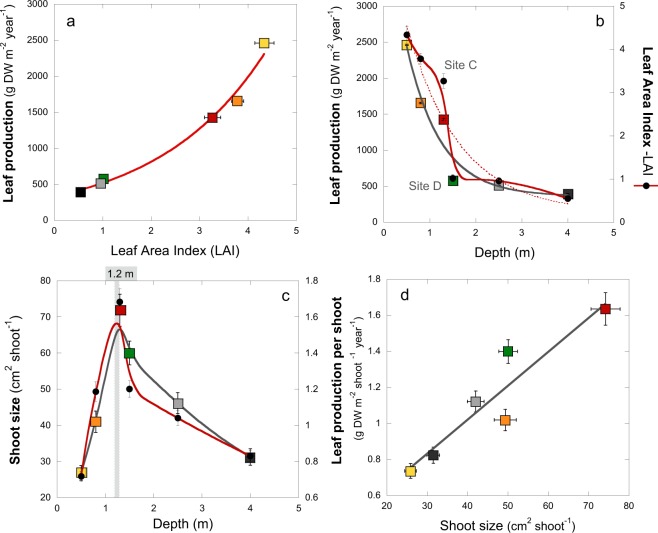
Seagrass leaf production. (**a**) Association between meadow leaf production (g dw m^−2^ yr^−1^) and LAI of *Thalassia testudinum* (R^2^ = 0.99; p < 0.001); (**b**) variation with depth in leaf production (white squares and black line) and LAI (black solid circles and red line), discontinuous red line is the exponential fit of the variation of LAI with depth; (**c**) variation of shoot size (black circles and red line) and leaf production per shoot (coloured squares and black line) with depth, the grey area highlights the peak estimated for shoot size and growth at 1.2 m depth; (**d**) association between leaf production per shoot (g dw shoot^−1^ yr^−1^) and shoot size (cm^2^ shoot^−1^), grey line represents a linear fit. See the text for an explanation of the line fitting shown.

Over the whole study, the coefficient of variation (C.V.) of all physiological parameters averaged 43%, ranging between 2.2% and 76.5% (Table 1). The lowest variability was found for *Fv/Fm* and the largest for leaf respiration. *Fv/Fm* showed the lowest values for site A and the highest for site B. Leaf pigmentation normalized to leaf area presented no significant pattern of change with depth, although site A showed lower values (SI-Table-S2). Leaf absorptance, however, varied significantly. The highest values were determined for sites B and D, whereas the lowest for site A (Table 1; SI-Table-S2). The photosynthetic descriptors differed significantly among sites and leaf sections (SI-Table-S3). Lower P_max_ was observed for sites A and C (Table 1) and towards the apical leaf sections (Fig. 4; SI-Table-S3). Photosynthesis was maximized in the middle of the leaf (portions “b” and “c”) and at intermediate depths (site D). The highest respiration was also recorded at intermediate depths while extremely reduced respiration was observed at site A (Table 1) and towards the apical leaf segments (SI-Table-S3). Photosynthetic efficiency (α) increased significantly with depth, although peaked at site D (Table 1). The lowest values were determined for site A, where significant differences between leaf portions “b” and “d” (SI-Table-S3) were observed. Compensation (*E*_*c*_) and saturation (*E*_*k*_) irradiances were consistently low for all sites (Table 1). *E*_*c*_ was significantly reduced at site A, and particularly towards the basal portions, while *E*_*k*_ peaked at site A and was slightly reduced at site C (Table 1). Significant increases towards the basal segments were also observed (SI-Table-S3). Estimates for *1/*Φ_*max*_ ranged between 12 and 16 mol quanta/mol O_2_ evolved (Table 1), showing no variation among sites and among leaf segments (SI-Table-S3), although a slight reduction was observed with depth.

### Leaf production

Leaf production was strongly correlated with LAI following an exponential function (Fig. 4a). Both parameters declined non-linearly with depth (Fig. 4b). Negative exponential functions described 86% of the variation of leaf production (p < 0.01) and LAI (p < 0.05) with depth. Two sites, C and D, differed from these quantitative descriptions of the variation of seagrass production and LAI with depth (Fig. 4b). The high leaf production and LAI observed at site C resulted from a large carbon allocation to shoot growth (Fig. 4c). A strong correlation between shoot size and leaf production per shoot was also found (r = 0.94; p < 0.01; Fig. 4d).

### Quantitative description

Based on the observed data, a unified quantitative model was constructed to give a continuous description of the morphological variation in shoot density and size, as a function of depth, for *Thalassia testudinum* in the lagoon of Puerto Morelos. This model was based on:

i) the non-linear decline in shoot density with depth described by Eq. (1) (Fig. 1d) and assuming a maximum of 2450 shoots m^2^ for shoot density (the maximum value determined);
ii) the bimodal variation of shoot size (Fig. 4c) described using two functions, a linear increase between A to C stations: Shoot size = 59.3 ± 7.6 * Depth (R^2^ = 0.99; p < 0.05); and a non-linear decline between C and F: Shoot size = 80.5 ± 12.5 * Depth^(−0.73±0.25)^ (R^2^ = 0.84; p < 0.01);
iii) and the linear association between shoot size and growth (Fig. 4d), for the estimation of the variation of shoot production according to the equation:

$${\rm{S}}{\rm{h}}{\rm{o}}{\rm{o}}{\rm{t}}\,{\rm{p}}{\rm{r}}{\rm{o}}{\rm{d}}{\rm{u}}{\rm{c}}{\rm{t}}{\rm{i}}{\rm{o}}{\rm{n}}=0.267\pm 0.17+0.0188\pm 0.004\ast {\rm{S}}{\rm{h}}{\rm{o}}{\rm{o}}{\rm{t}}\,{\rm{s}}{\rm{i}}{\rm{z}}{\rm{e}}\,({{\rm{R}}}^{2}=0.87;{\rm{p}} < 0.01)\,$$

**Figure 5:**
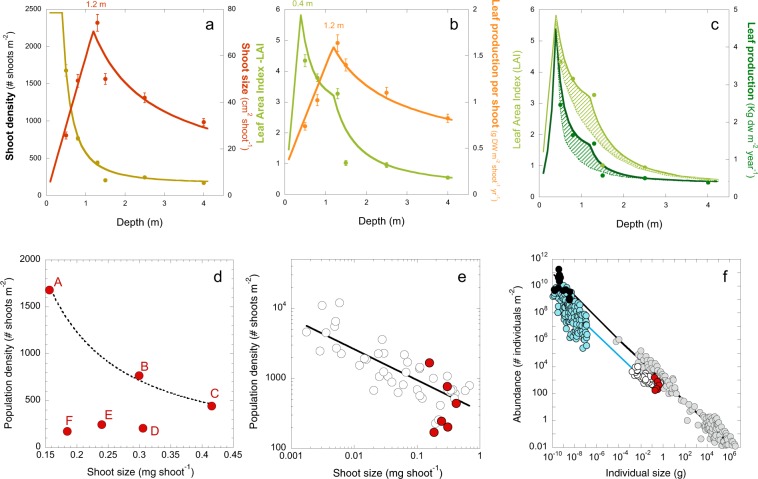
Quantitative description of seagrass changes and plant self-thinning responses. Quantitative description: (**a**) Quantitative description of the variation in shoot density (light brown) and shoot size (red) with depth; (**b**) quantitative description of the variation in LAI (light green) and leaf production per shoot (orange) with depth; (**c**) quantitative description of meadow production (dark green) and LAI (light green) with depth, the dotted lines illustrate the theoretical result if shoot size were constant, hence the shaded area represents the increase in meadow production and LAI due to the shoot size response. In all plots (**a**–**c**) circles describe the avg ± SE empirical values and the continuous line the quantitative description according to the model. For the description of the self-thinning response: (**d**) illustrates the co-variation of shoot size and shoot density for the six meadows characterized, letters refer to each site (from A-shallowest to F-deepest); (**e**) association between shoot size and shoot density for 14 seagrass species with different size, in red are highlighted the values estimated for *Thalassia testudinum* in the present study; and (**f**) comparison of seagrass data (white and red circles) against values from Belgrano *et al*.’s^36^. for terrestrial plant communities (grey) and phytoplankton (blue and black circles). Black circles describe maximum mass achieved by phytoplankton. Lines illustrate the different fits determined for: (**d**) the variation of three sites; (**e**) the variation of 14 seagrass species; and (**f**) black, the variation of terrestrial plants; (**f**) blue, the variation of aquatic organisms (submerged plant canopies and phytoplankton).

Estimated values for the model fitted the empirical data well (Fig. 5a). The model highlighted a peak in shoot size at 1.2 m depth (Fig. 5a), and a peak in LAI at 0.4 m depth (Fig. 5b). The shoot size peak coincided with a peak in shoot growth (Fig. 5b). The predicted changes in LAI as a function of depth were used to estimate continuous changes in meadow production with depth (Fig. 5c). The model highlights the interpretation that reductions in shoot growth are the determinant for the reduction in LAI and shoot production below 1.2 m depth, and that the high seagrass production observed on the platform was facilitated by the increase in shoot growth (Fig. 1). A comparison of the actual LAI and leaf production values with theoretical predictions assuming no increases in shoot growth illustrates the effect of this response on seagrass production and LAI (Fig. 5c).

### Seagrass self-thinning

The simultaneous occurrence of an increase in shoot growth and a reduction in population density was only observed on the seagrass platform (sites A-C, Fig. 5 plots a and d). Below 1.2 m depth, shoot density and size were, both, progressively reduced. As a comparison, data on population densities and shoot sizes from 14 seagrass species covering a wide range of plant sizes was collated (Fig. 5e). This data indicates a general pattern in seagrasses where shoot size decreases as population density increases (R^2^ = 0.52; p < 0.01), which is consistent with the within-species variation observed in this study. When comparing seagrass data against a more general description for terrestrial plants and phytoplankton^36^, a similar allometric scaling-factor (log-log slope, ANCOVA, slope t-test, p > 0.05; see Fig. 5f) was found for all photosynthetic organisms. It is remarkable that the maximum mass achieved by aquatic organisms, seagrasses and phytoplankton is significantly lower than that of terrestrial plants (ANCOVA, intercept t-test, p < 0.05) despite both groups lie on the same general trend.

## Discussion

### Depth acclimation response of Thalassia testudinum

The results indicate that carbon allocation to different plant components governs the response of *Thalassia testudinum* to depth. That is, the response is largely a change in canopy structure and belowground biomass rather than in leaf physiology. The significance of structural changes in the response of large seagrasses to depth has been previously indicated^19–21^, whereas larger variation in leaf physiology characterizes the environmental response of smaller species^19,29^. These observations have been attributed to the capacity of large seagrass species to modify the light environment within the canopy, but this hypothesis does not explain the low physiological variability found for the leaves of *Posidonia oceanica* in their acclimation to depth^19^. Our study measured at the centre (middle level) of the canopy of *T. testudinum* and for canopies placed at different depths and with different physical structures, similar internal light fields. This canopy level corresponded with the location of the most photosynthetically active region of the leaf. Therefore, the variation of the light environment within the canopy is consistent with the low physiological variability found for the leaves of *T. testudinum*. However, we also observed consistently low values for saturation (*E*_*k*_ < 100) and compensation (*E*_*c*_ < 25) irradiances, and the minimum quantum requirements of photosynthesis (*1/*Φ_*max*_) was in general close to the theoretical minimum of 8 photons absorbed per O_2_ molecule evolved^37^. These results agree with the previous conclusion that the leaves of *T. testudinum* are shade-adapted, in the Darwinian sense^15^. Indeed, this shade-adapted condition could explain why the maximum quantum yield of photosynthesis (Φ_*max*_) of the leaves of this tropical seagrass was not influenced by depth, as it is already close to maximized in low conditions. According to these results, the structural adjustments documented for the canopy in response to depth were fundamental to maintain a homogeneous light environment for the leaves along the depth gradient investigated (0.5–4 m). The maintenance of low light levels within the canopy is particularly important at the shallowest sites^14^ since the shade-adapted leaf physiology of this species constraints its capacity to acclimatize to high light^15^. Our results suggest that the ecological and evolutionary success of this important habitat-builder in the shallow and highly illuminated reef environments of the Caribbean is reliant on the morphological plasticity of the canopy and its capacity to modify the light environment of the leaves.

### Structural response of the canopy

Changes in LAI and shoot density were the best descriptors of the magnitude of leaf self-shading within the canopy, measured here in terms of the light attenuation coefficient of the canopy, *K*d_*canopy*_. Increases in shoot size also contribute to increase *K*d_*canopy*_, as larger shoot size ad shoot density together increase self-shading, as previously documented^33^. The quantitative model proposed by Enríquez & Pantoja-Reyes for *T. testudinum*^33^ and supported by our new characterizations, enables prediction of the effect of canopy changes on the magnitude of leaf self-shading within the canopy (i.e., *K*d_*canopy*_ variation). The model encapsulates the explanation for the dominance of these morphological/structural changes in the depth response of *T. testudinum*. Reductions in shoot density and LAI with depth allow “opening” the canopy, facilitating light penetration and maintaining leaf-level photosynthetic activity. In contrast, the high LAI values and shoot densities observed at the shallowest sites, “close” the canopy, providing shade and photoprotection^25^ to a shade-adapted leaf physiology under what would be otherwise damaging high-light conditions^14,15^. Reductions in shoot density, shoot mass, and the number of leaves per shoot have been widely documented for seagrass meadows^19,20^, whereas LAI is less frequently characterized. Declines in LAI with depth have only been reported for two species^30^. The low attention that LAI has received in marine research contrasts with the significance of this parameter in terrestrial ecology, where LAI is considered the primary descriptor of light interception and leaf production of plant canopies^38,39^. To our knowledge, this is the first time that both functional attributes have been demonstrated in terms of LAI for submerged plant canopies. According to them, LAI also plays a fundamental role in the structural and functional characterization of marine ecosystems, and we recommend this parameter as a key metric for future studies.

### Self-thinning response

**Figure 6:**
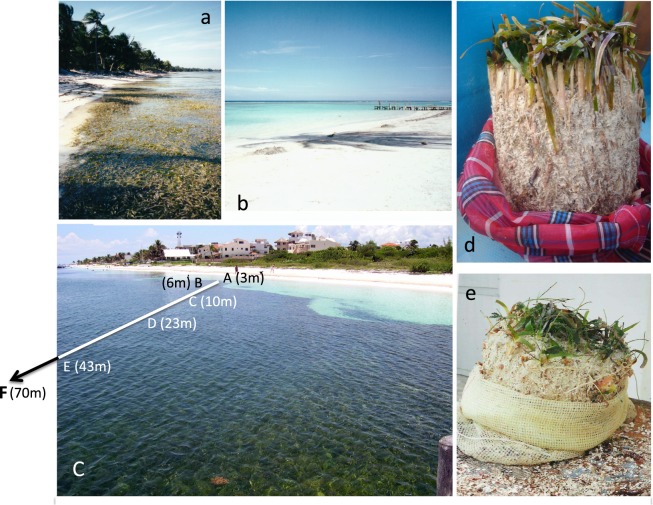
Images of the waterline of Caribbean reef lagoons, with and without seagrass presence, and of two core samplings. (**a** and **b**) Images with and without the presence of the seagrass at the shore; (**c**) the meadow characterized in this study, indicating the relative position of the five sites investigated (not to scale); and (**d** and **e**) two cores sampled within the seagrass platform showing the underground seagrass biomass and carbonate sediment. The white colour indicates the low sediment content of organic at the time of sampling.

The highest LAI and leaf production occurred on the coastal platform constructed by *T. testudinum* close to the shore (Figs 1 and 6). In these meadows, as depth increased, we observed an increase in biomass allocation to shoot growth coincident with the decline in shoot density. The simultaneous occurrence of both processes, a reduction in population density and an increase in biomass per individual (shoot), has been traditionally considered a “plant self-thinning” response^40–42^. However, this simultaneous variation was only observed on the coastal platform. For depths beyond the peak of shoot size (here found at 1.2 m depth), shoot density and size were both reduced with depth. This observation may draw into question the occurrence of plant self-thinning in seagrasses, but an interspecific comparison (Fig. 5e,f) revealed similar allometric scaling-factors for the self-thinning response of seagrasses, terrestrial plant canopies and phytoplankton communities. Therefore, our study supports the manifestation of the self-thinning response in seagrasses, but, furthermore, the hypothesis of a general law across photosynthetic structures, from unicells to complex terrestrial and submerged canopies of higher plants^36^. This law establishes a limit/threshold for the maximum biomass that the photosynthetic organism or the community can “pile up” (i.e., photosynthetic mass per projected area). Aquatic photosynthetic structures (phytoplankton and seagrass canopies) seem to present higher structural constraints than terrestrial plants, as indicated by their lower biomass maxima (Fig. 6f). The different physical constraints of building photosynthetic canopies/communities in air and water may play a role to explain these differences. Intraspecific competition has traditionally explained plant self-thinning in terrestrial plants^41,42^, and more recently, it has been also suggested the importance of multiple agents such as pathogens, insects and mammals^43^. However, our study unveiled a critical role of light attenuation within the water column and pigment self-shading within the canopy for the understanding of the structural variation of the seagrass canopy, and for explaining the “disappearance” of the plant self-thinning response below 1.2 m depth. Therefore, light limitation of photosynthetic activity due to pigment self-shading could explain why all photosynthetic organisms are affected by a similar physical constraint. This interpretation may also explain why changes in leaf area are better descriptors of plant self-thinning than changes in plant weight^44^, and some of the exceptions (i.e., shaded species) documented for the self-thinning rule^45^.

### Transition between vertical and horizontal seagrass growth

The limit for the seagrass self-thinning response with depth, which ends the progression of the seagrass platform, was found to be associated with a switch in mass allocation from vertical growth (shoot extension) to horizontal growth (shoot recruitment). The two sites located across this transition, C and D, had values for LAI and leaf production significantly different to those predicted by the quantitative model developed above (Fig. 5b). The situation at site C could be explained as the limit of the seagrass self-thinning process, but the lower seagrass biomass observed at site D indicates insufficient seagrass colonization, due perhaps to higher local disturbance and/or equivalently lower seagrass ability to stabilize sediments when belowground mass and vertical growth are reduced. While this study can only offer potential hypotheses for explaining this transition, what is remarkable is that our analysis revealed the differences between these two sites, separated by only 13 m, throughout an apparently fully colonized seagrass meadow. The model also predicted the location of the depth limit for the progression of the seagrass platform in the reef lagoon of Puerto Morelos at 1.2 m depth and at a distance from the shore around 10–23 m. It seems reasonable that this quantitative model may be a useful reference for the maximum seagrass mass that could be expected in this particular reef lagoon at full seagrass colonization (i.e., seagrass spatial variation with depth). Such characterization of the maximum seagrass abundance may also describe the variation of other Caribbean reef environments with similar water transparency, nutrient conditions and temperature and salinity regimes. Moreover, in cases of physical or anthropogenic disturbance, this quantitative description may be useful as a reference for interpreting consequential variation in seagrass mass, carbon allocation, LAI and seagrass production. The model may also be fundamental for constraining the output of remote sensing analyses of depth and LAI^46^. The identification of the depth for maximum shoot growth also allows quantification of the coastal area where seagrass production is likely to be maximized.

### Construction and ecological value of the coastal seagrass platform

The ecological benefit of the self-thinning response for this seagrass was manifested in the significant increases in LAI and leaf production observed on the coastal platform, 2.5 to 6 times higher production than at the deepest sites, and despite we measured there the lowest irradiance at the lowest levels of the canopy. Only a shade-adapted leaf physiology could ensure enough photosynthetic activity at the lowest levels of dense canopies. Therefore, the maintenance of high LAI in addition to the persistence of a shade-adapted leaf physiology, were both fundamental to maximize seagrass production on the dense coastal meadows. The seagrass platform developed at the shore represented just a thin coastal fringe (<20 m) only present in certain areas of this reef lagoon at the time this study was performed (Fig. 6). Its construction requires the accumulation of dense stands of seagrass belowground mass and sediments, as illustrated in plots e and f of Fig. 6. We estimated values for the seagrass belowground biomass on the platform above 740 g dw m^−2^ (Fig. 1), which supports the ability of the turtlegrass to retain organic “living” carbon and sediment in tropical habitats^3^. *Thalassia testudinum* has shown extraordinary ability to increase vertical (shoot) growth in response to large volumes of sediment migration^9^, whereas its capacity to re-populate an empty area through favouring horizontal extension (shoot recruitment) is reduced in oligotrophic reef environments due to nutrient limitation^13^. The widespread observation of grass-free depressions (“blowouts”) within these meadows^47,48^, stresses the importance of disturbance and hydrodynamics in the formation and persistence of the dense coastal seagrass platform. Therefore, the frequency and intensity of physical disturbances^7,49^ as well as sediment accretion and species ability for sediment trapping and stabilization, will explain the duration of the construction and the persistence of a seagrass coastal platform in a particular habitat. In the case of *T. testudinum* reef habitats, the reduced vegetative growth of this species in oligotrophic reef environments^11–13^, suggests that sexual reproduction (seedling settlement) may also play an important role in the formation of the coastal platform, as supported by the larger diversity of genets documented for these coastal meadows in the reef lagoon of Puerto Morelos (mean-largest-genet = 10.3 m) versus the deepest sites (mean-largest-genet = 167.3 m)^50^.

### Relevance of belowground biomass variation in the photoacclimatory response

The shade-adapted leaf physiology and the low quantum requirements of photosynthesis determined here and elsewhere for *T. testudinum*^15^ suggest that this important habitat-builder in the Caribbean and Gulf of Mexico may have the ability to colonize deep areas and cope with significant reductions in irradiance. However, a quite shallow maximum depth distribution has been documented for this species in clear tropical waters (~10–12 m^51,52^), and sparse canopies rarely occur at depth. Moreover, *T. testudinum* has also shown high vulnerability to severe reductions in light availability (die-offs)^53^. To explain this apparent contradiction, it is important to remark that seagrass belowground biomass often represents in reef habitats the largest component of the whole-plant mass of *T. testudinum* (80–90%; cf.^33,47^). This mass allocation leads to substantial increases in whole-plant respiration^30^ and, hence, in the minimum quantum requirements of plant growth-MQR^15^. Thus, the reduction observed in the belowground mass with depth has to be considered a fundamental component of the seagrass acclimatory response to decreasing irradiances, to facilitate adjustments in whole-plant respiration in line with the decline in canopy photosynthesis.

### Ecological implications

Sparse-deep meadows developed a more diverse co-habiting macrophyte community than dense-shallow beds. In particular, the pioneer seagrass species, *Syringodium filiforme*, increased in abundance in the abrupt transition between sites C and D, exactly where the climax species, *T. testudinum*, presented incomplete colonization. Hydrodynamics and nutrient competition have been considered the primary environmental drivers of the spatial macrophyte diversity observed in Caribbean reef habitats^54^. We do not question this interpretation but our results also suggests the important role of light and the consequential structural adjustments of the habitat-builder to depth, to explain the natural variability observed in the macrophyte community.

The exponential reduction with depth of the belowground biomass of *T. testudinum* may affect the capacity of this habitat-builder to retain organic carbon, as carbon retention is restricted in in reef habitats to the structural seagrass “living” carbon (underground biomass). The low capacity of *T. testudinum* to retain the large amounts of organic matter produced in coral reef environments has been widely documented^8^, and confirmed for the reef lagoon of Puerto Morelos at the time this study was performed, in view of the low organic content (<3%) of the coarse carbonate sediment (^55^ and Fig. 6e,f). So, we can conclude that habitat capacity to store blue carbon in Caribbean reef environments is significantly reduced with depth associated with the exponential reduction in seagrass mass [22 and this study]. In practice, the contribution of *T. testudinum* to sequester organic carbon across pristine Caribbean reefs is probably restricted to a small coastal fringe with variable width (<20 m), corresponding to the coastal seagrass platform limit. Further, this dense seagrass fringe is present only when the frequency and severity of physical disturbances are low enough to allow its slow development and persistence. When present, these coastal meadows may represent a significant blue carbon reservoir. Unfortunately, we still known very little about the dynamics of construction and erosion of the seagrass platform developed by *T. testudinum* at the shore. However, we offer here its first characterization and also a mechanistic basis / hypothesis to explain the environmental regulation of this structural variation.

In a global context, our study demands a re-evaluation of current estimations of the contribution of *Thalassia testudinum* to sequester carbon in Caribbean reef environments. Smaller species like *Syringodium filiforme, Halodule wrighti, Halophila spp*. cannot exhibit similar capacities to retain organic carbon in comparison to habitat-engineering species such as *T. testudinum*, in consonance to a similar analogy between grasses and trees in terrestrial plant communities. Considering the current environmental crisis derived from the massive arrivals of pelagic *Sargassum spp*. to Caribbean coral reef environments^56^ and their accumulations at the shoreline^57^, these potential coastal reservoirs of blue carbon are particularly threatened.

Bearing in mind the worldwide degradation of seagrass habitats^58^, the quantitative descriptions of maximum seagrass abundance in combination with remote sensing techniques provide powerful tools for the interpretation of spatial and temporal changes in seagrass mass and LAI^46,59^, strengthening our capacity of prediction and diagnosis of habitat condition. These characterizations facilitate recognition of disturbed sites, and also evaluation of the success of seagrass restoration programmes, as well as prediction of the impact of incomplete seagrass colonization on leaf production and habitat carbon storage capacity.

## Conclusions

Two “self-pruning” structural responses govern the photoacclimatory response of *Thalassia testudinum* to depth: “canopy opening”, to facilitate light penetration and maintenance of sufficient illumination at leaf surfaces; and “below-ground biomass depletion”, to maintain plant carbon balances by compensating for decreased canopy photosynthesis. These structural changes performed by the main habitat-builder enhance macrophyte diversity with depth, while habitat capacity to sequester carbon diminishes. The ability to construct complex canopies and regulate the internal light field may be key for the survival of *T. testudinum* at depth, and also for explaining the coastline colonization in well-illuminated reef habitats. Dense coastal meadows have maximal carbon storage capacity thanks to a self-thinning response, which becomes limited by light at a certain depth, possible in conjunction with a transition from a vertical to horizontal growth pattern. Despite the complexities of the aquatic environment, *Thalassia testudinum* and seagrasses adhere to a universal allometric-law that describes the self-thinning plant response, and is consistent from unicells to complex plant canopies, both terrestrial and marine.

## Methods

The study was conducted in June-July 2004 in a continuous, non-patchy, meadow located in a fringing-reef lagoon in the Mexican Caribbean (N:20°51.4′ y W:86°52.251′). Six stations (A–F) were selected along a 70 m length transect perpendicular to the shoreline, following a 0.5–4 m depth gradient. The distance between stations increased non-linearly to more evenly distribute the expected variability (Fig. 6c). The transect was positioned on a well-established meadow until hurricane Wilma impacted the area in October 2005.

Instantaneous irradiance was measured *in situ* at each site, following Enríquez & Pantoja-Reyes^33^. Downwelling irradiance was measured at solar noon, within the water column and along 6 to10 canopy profiles, integrating 60 s at every centimetre. All determinations were performed in clear days (no wind and cloud cover), which allowed comparable measurements among sites under maximal water clarity and minimal sediment re-suspension^33^. Based on these profiles, we calculated the downwelling attenuation coefficient for the canopy (*K*d_canopy_, m^−1^) and the downwelling attenuation coefficient for the water column (*K*d_water_, m^−1^).

### Meadow diversity, leaf production and plant structure

Species composition, seagrass shoot density and total above-ground biomass of the macrophyte community were evaluated on six replicates of 0.08 m^2^ square area at each site. Morphological seagrass descriptions were performed using small cores (12 cm diameter), collecting ~20 intact vertical shoots at each sampling depth, and using a 30 cm diameter core (n = 4) to obtain complete plants with intact rhizomes and roots. Belowground biomass and above/belowground ratios were determined with this core. Only living tissue was considered for the estimation of biomass partitioning.

Leaf growth was determined in triplicate 0.25 m^2^ sampling areas following the modified leaf marking technique^60^. All the leaves inside the sampling area were marked just above the sheath. After 10 days the same leaves were marked again and harvested for growth determinations. Harvested shoots were gently cleaned removing the epiphytes with a razor blade. For each shoot we determined: the number of leaves, the total length and width of each leaf, the length of the new segment, and the length of the new leaves. The leaf material was dried at 60 °C for 24 hours to determine total dry weight (dw). Shoot leaf production (gdw shoot^−1^ day^−1^) was calculated by dividing by the number of days lapsed between first marking and sampling. Meadow production (gdw cm^−2^ year^−1^) was calculated normalizing for shoot density, and extrapolating to annual production assuming a constant leaf growth over the year, which is unrealistic but facilitates comparison with other studies. Our estimates fit in the range of previous studies^47,61^.

Shoot size (total one-sided-leaf-area), number of leaves per shoot, maximum leaf length and width were determined on the ~20 intact vertical shoots collected at each site. The leaf area index (LAI) was estimated as the ratio of the total one-sided-leaf-area to ground area, using the quadrat area as a ref.^33^.

### Leaf physiology

Leaf physiology was characterized at four levels along the second-youngest leaf, from apical (portion-“a”) to basal (portion-“d”). Typically, the second-youngest leaf is the best choice to characterize the photoacclimatory response of *T. testudinum*^14,15,25^, as it has already achieved the maximum length and developed the photoacclimatory response along the within-canopy light gradient, with still minimal accumulation of epiphytes and damage. Leaf fragments were only selected from the mature blade, excluding the immature basal segment according to Enríquez *et al*.^14^ and Schubert *et al*.^25^.

Photosynthesis vs. Irradiance (*P vs E*) curves were obtained with polarographic oxygen Clark-type electrodes in water-jacked chambers (DW3, Hansatech, UK) following the protocol described by Cayabyab & Enríquez^15^. We calculated: photosynthetic efficiency (α, µmol O_2_ µmol incident quanta^−1^) from the initial slope by linear least-squares regression analysis; maximum photosynthetic rates (*P*_*max*_, µmol O_2_ cm^−2^ h^−1^) from the average values above saturating irradiance; light-enhanced respiration (R_L_), in darkness, right after the leaf segment had reached *P*_*max*_; saturation irradiance (*E*_*k*_) as *P*_*max*_/α; and compensation irradiance (*E*_*c*_) as the intercept with the irradiance axis. These characterizations were performed for four replicates (n = 4) per leaf section and station.

Light absorption was determined spectrophotometrically as absorbance (D) using the methodology described by Enríquez^62^ and Vásquez-Elizondo *et al*.^63^. Absorptance (A), defined as the fraction of incident light absorbed by leaf pigments, was calculated as: A = 1–10^−D^ − R, where R is leaf reflectance. Optical determinations were performed on the same fragments used for the physiological characterizations. Half of the segment was frozen (−70 °C) for pigment analysis right after the optical measurement. Chlorophyll content was determined according to Cayabyab & Enríquez^15^. Finally, we also estimated for each sample the minimum quantum requirements of photosynthesis (*1/*Φ_*max*_; quanta absorbed O_2_ evolved^−1^) from the inverse of the quantum yield of leaf photosynthesis (Φ_*max*_), after absorptance correction (α/A).

### Statistics

All results are expressed as mean ± standard error (SE). ANOVA-one-way tests and Factorial ANOVA-two-way tests were used to detect significant differences among sites, and the effect of depth and/or leaf segment. A Tukey-HDS-post-hoc test was also applied. Normality and homoscedasticity were tested using, respectively, Shapiro and Levene’ tests. When data failed the parametric assumptions, the p-value was lowered from 0.05 to 0.01 to minimize the risk of a Type 1 error^64^. We also used non-linear models (power- or exponential-functions) to describe the variation with depth. Co-variance analyses were used to detect differences in the allometric functions (power-functions), using log/log-transformed data.

Data were analysed using R-software^65^.

## Supplementary information

Tables S1 to S3

Figure S1: Photosynthesis vs Irradiance curves

## Data Availability

All data generated or analyzed during this study are included in this published article (and as supplementary information). Any more detailed information will be available from the authors on reasonable request.
