# Supplementary material for: Structural complexity governs seagrass acclimatization to depth with relevant consequences for meadow production, macrophyte diversity and habitat carbon storage capacity
Source: Sci Rep. 2019 Oct 10;9:14657. doi: 10.1038/s41598-019-51248-z (PMC6787196; doi:10.1038/s41598-019-51248-z)
Supplement: Supplementary file 2 — Figure S1: Photosynthesis vs Irradiance curves [file 41598_2019_51248_MOESM2_ESM.pdf]

Structural complexity governs seagrass acclimatization to depth with relevant consequences for meadow production, macrophyte diversity and habitat carbon storage capacity

Susana Enríquez, Irene Olivé, Napo Cayabyab, and John D. Hedley

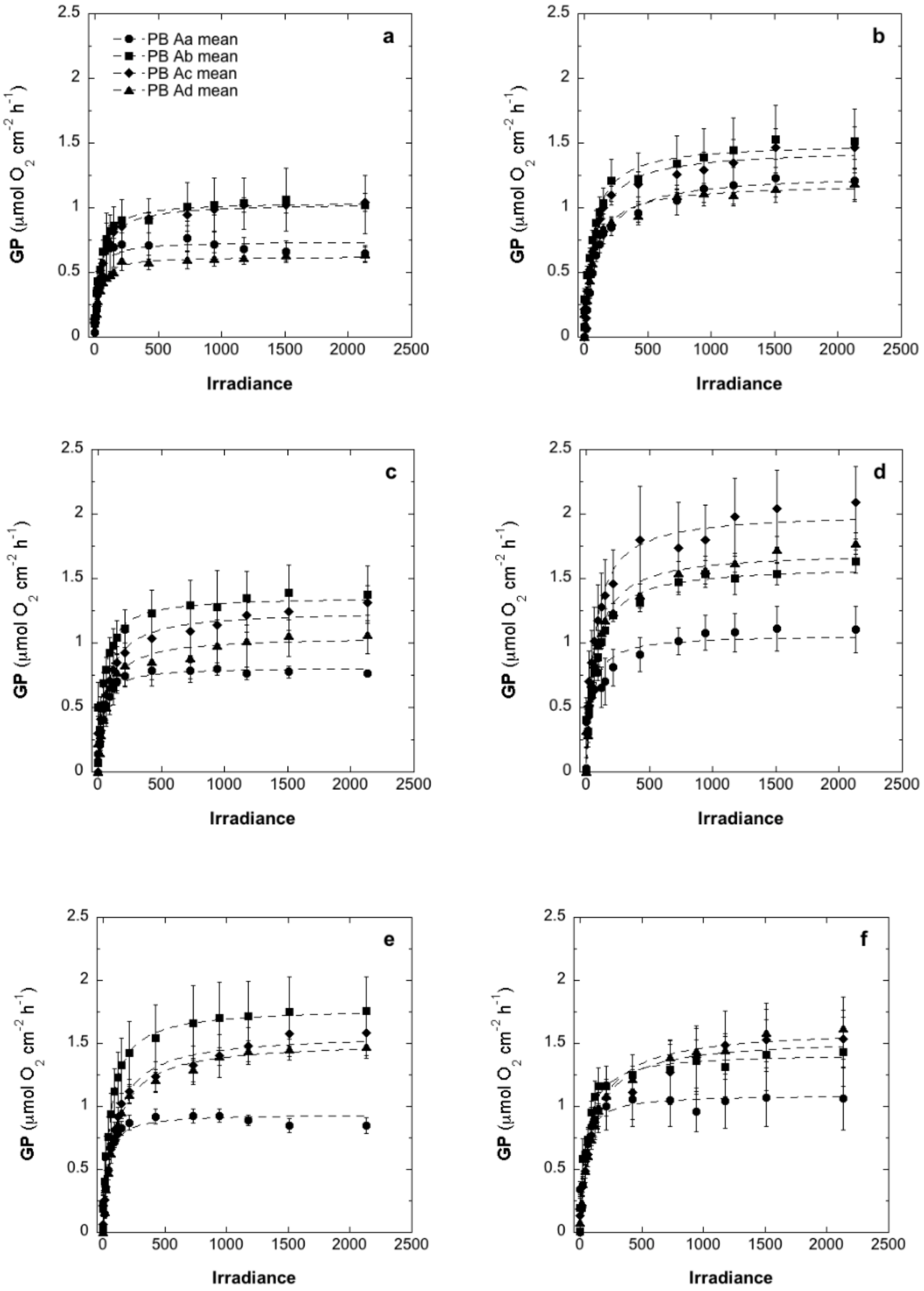

Figure S1: Photosynthesis vs. Irradiance curves (P vs E) for four leaf segments (a-d) located at different positions within the seagrass canopy, from a-apical (circle) to d-basal leaf segment (triangle). Each plot (a-f) corresponds to the same site label (A-F) located along a continuous seagrass meadow, from the shallowest (A) to the deepest (F).
